# The microbiome and the human: A reply to Parke and colleagues

**DOI:** 10.1371/journal.pbio.2006974

**Published:** 2018-09-12

**Authors:** Tobias Rees, Thomas Bosch, Angela E. Douglas

**Affiliations:** 1 Berggruen Institute, Los Angeles, California, United States of America; 2 Zoological Institute and Interdisciplinary Research Center Kiel Life Science, University of Kiel, Kiel, Germany; 3 Department of Entomology, Cornell University, Ithaca, New York, United States of America; 4 Department of Molecular Biology and Genetics, Cornell University, Ithaca, New York, United States of America

We are grateful to Parke and colleagues [1] for their comment on our recently published essay [2]; it provides a much welcome opportunity to clarify our argument. Parke and colleagues offer two different kinds of critiques.

The first kind is specific to biology and comes in two iterations. (i) Parke and colleagues are concerned that the reference to coconstitution in our essay might be indicative of coevolutionary processes, according to which either the microbiome or the human plus the microbiome is a “unified entity.” This illustrates a common misunderstanding: developmental and physiological integration (or coconstitution) does not necessarily imply or require that the human and its microbiome comprise a single evolutionary unit. We trust that it is evident from the context that our reference to coconstitution relates to physiological processes. (ii) Parke and colleagues worry that “suggesting the whole microbiome is causal is problematic.” We agree entirely with Parke and colleagues. There is an abundance of evidence that individual taxa vary in their impact on the traits and fitness of the host and that the magnitude of this variation can depend on host genotype, environmental factors, and so on. The absence of strict partner fidelity is fully compatible with our statements that key aspects of human function traditionally treated as defining the individual “would not function without their microbes” and that we and our microbes are “physiologically and developmentally united”.

The second kind of critique concerns philosophy. Parke and colleagues take issue with what they identify as our unsystematic use of the term self. More specifically, they critique us for not sufficiently differentiating the ways in which the concept of self is used in the natural and the human sciences and how the microbiome and its impact on biological definitions of self influence each of the definitions of self in the human sciences. As they see it, our blurring of these two broad fields of research amounts to a category mistake insofar as we seek to answer a philosophical question (“what does it mean to be human?”) by attending to the biological (“the specifics of our biological constitution”). According to Parke and colleagues, our humanity doesn’t emerge from our biology but instead from “who we are as self-cognizing individuals, with our rich diversity of mental, emotional, and cultural resources.” It appears that they are suggesting, however implicitly, that the human (self-cognizant or, more classically, constituted by reason) and nature (not self-cognizant and reducible to mere matter) are qualitatively––ontologically––different realms; Parke and colleagues juxtapose our “biological constitution” or “the material basis of our mind” with our “rich diversity of mental, emotional, and cultural resources.” It is here that we disagree.

What we find thought provoking about microbiome research is that it cuts across just this classical distinction between the human here and nature there. If it is impossible to disentangle a human brain, genome, or immune system from the workings of the microbes that live in and on us, then how can the sharp dualism between humans and nature be maintained? Of course, the human brain and microorganisms are separate entities; in terms of their cellular identity, they are different. However, in terms of their physiological function, they are partly integrated. If one thinks from the perspective of this partial integration, then the idea that humans are more than mere nature that has organized the classical division of labor between the faculty of arts (concerned with the realm that opens up beyond nature) and the faculty of science (concerned with the mere nature) becomes difficult to maintain. Our point is not to call into question the human sciences. On the contrary, our point has been to call into question the conception of the human and of nature that has made the distinction between the natural sciences and the humanities plausible in the first place. This leads to the priority of building a venue of research in which those questions that were formerly left to the natural sciences and the human sciences are not mutually exclusive.
